# Therapeutic Notes

**Published:** 1896-01-25

**Authors:** 


### THERAPEUTIC NOTES.

### MORPHINE AND OPIUM.

**Morphine Sickness.**—The nausea and vomiting which constitute a practical difficulty in taking opium preparations are discussed by Guinard.[1] He found that 60 per cent. of dogs narcotised by morphine vomit before going into morphine sleep. Trousseau and Bonnet showed long ago that the method of administration is not without influence, and that if vomiting occurred rapidly after endermic injection it is slow when given by the mouth. This does not seem in favour of a peripheral origin of the vomiting. Guinard proved that it was not peripheral by section of both vagi, which did not prevent nausea and vomiting after injection of weak doses. These experiments exclude the possibility of stomach reflex vomiting by irritation conveyed through the vagi. He affirms that the essential cause of morphine vomiting is found in an immediate irritation of the vomiting centre, the first doses of the drug carried by the circulation into contact with the vomiting centre, and is exactly the same mechanism as in the case of the vomiting produced by apomorphine. Large doses of morphine are less likely to cause vomiting than small doses. It is exceptional to see vomiting in dogs following intravenous injections of morphine, which bring the drug very rapidly in quantity into the organism; when absorption takes place slowly, vomiting is more likely to occur. It is an illustration of contrary actions; a drug which in weak doses acts as a stimulant to the centres, on which it exerts an elective action, has a paralysing effect when it reaches these centres in larger quantity. He mentions as remedies which can be used against the production of the exciting effects of morphine on the vomiting centres chloroform, ether, chloral. Brown-Séquard pointed out that atropine, added to the morphine, prevented vomiting in the majority of cases. If this be true in man, Guinard has not found it so in dogs, atropine assisting rather than preventing the vomiting resulting from morphine.

**Antidotes in Morphine Poisoning.**—J. W. Stickler[2] has recorded results of investigations into the antagonism between cocaine and morphine, a subject of first importance. His experiments were conducted on pigeons, dogs, and man, and he adds some clinical observations which greatly enhance the value of his contribution. He also investigated the effect of atropine in combating the action of cocaine. A comparison of his results showed that, in relation to pigeons, and taking into account all the birds treated, those having first morphine, then a lethal dose of cocaine (determined by controlled experiments) had an average life of 44 min. 8 sec.; those having first atropine, then a lethal dose of cocaine, had an average life of 1 min. 42 sec.; those having only a lethal dose of cocaine had an average life of 1 min. 2 sec. This comparison shows conclusively, so far as figures are concerned, that morphine and cocaine are manifestly antagonistic to each other. It further shows that atropine furnishes almost no protection (probably none) against the prompt and violent action of cocaine. As far as the dogs are concerned, it would seem that while two grains of morphine do not successfully antagonise one grain of cocaine, four grains of morphine will serve as an antidote for four grains of cocaine. A comparison of results furnished by the investigations upon the man clearly shows the mutual antagonism between the two drugs. Morphine contracted the pupils, cocaine dilated them; morphine retarded the pulse-rate somewhat, but did not alter its force perceptibly, cocaine accelerated the pulse and strengthened it; morphine diminished the number of respirations to the minute, respirations were increased in number by cocaine (though when any considerable quantity of cocaine is taken respirations are fewer per minute). Morphine produced a sense of muscular fatigue and drowsiness, cocaine energised, and had a waking-up effect upon the intellectual faculties; morphine caused dryness of the throat, mouth, and skin, also impaired functional activity of the kidneys, while cocaine increased the moisture of the throat, mouth, and skin, and increased the functional activity of the kidneys. Thus it is seen that those two agents are opposed to each other in their therapeutic action upon man. Stickler states that he has given to patients who have had severe pain ¼ gr. of morphine and ¼ gr. of cocaine hypodermically, and not only has the pain been relieved, but there has been no drowsiness, nausea, or vomiting to interfere with attention to the business of the day. When morphine is to be given at night in comparatively small doses, it is best not to use cocaine, as it is likely to prevent sleep, so decided is it in its effect upon the brain. He further quotes a case demonstrating the value of cocaine as an antidote for opium. Dr. Matthews, his associate in laboratory work, was called to Mrs. R——. About a quarter to seven p.m. she took 2½ oz. of laudanum. When seen twenty minutes later the pupils were contracted, but she was greatly excited. He produced emesis by six grains of hydrarg. sulph. flava. but the vomit had neither the smell nor appearance of laudanum, and the doctor concluded that most of the laudanum was absorbed. He injected ½ gr. of cocaine. At twenty minutes to eight she was quieter and felt more vigorous; he then gave ¼ gr. cocaine, and in one hour she was perfectly normal and in her general condition, her pupils being well dilated. Stickler doubts if permanganate of potash or atropine would have given as good results as this.

In conclusion, the author states that in any case of opium poisoning he would first employ an emetic, then would give hypodermically a quarter to half a grain of cocaine; wait twenty minutes, and if no decided effect had been obtained he would give another injection of a quarter grain. After waiting another twenty minutes he would repeat the dose if there were no manifest improvements in the case. He thinks three separate doses of a quarter grain each at intervals of twenty minutes is the best plan to follow, on account of the very quick diffusibility of the drug and its sustained effect. During this time he would administer coffee by mouth or rectum as a supplementary heart stimulant, and in extreme cases employ artificial respiration.

**Permanganate of Potassium as an Antidote in Opium Poisoning.**—We are indebted to William Moor for bringing forward the usefulness of this drug in opium poisoning, but though he urged its claims so far back as 1894, its utility is still the subject of difference of opinion in the profession. Louis Sharp[3] recalls Moor's experiments with morphine, permanganate, and egg albumin, in which he (Moor) claimed showed that one grain of permanganate will destroy one grain of morphine, even in the presence of other organic matter. He therefore inferred that there was such an affinity between the two drugs that, if the permanganate is brought into contact with organic matter containing morphine, it will act on the morphine first. He experimented on himself, taking three grains of morphine by the mouth, followed immediately by four grains of permanganate, with no unpleasant results. Sharp refers to the observations of others, whose experiences corroborate or controvert Moor's conclusions.

Now, Moor[4] himself has restated his views thus: "Permanganate of potassium . . . intrinsically cannot possibly be inefficient in antidoting morphine. One may just as well state that a strong alkali is incapable of neutralising a mineral acid"; and he gives the following directions for its administration: (1) One grain of permanganate of potassium decomposes one grain of morphine (six grains of opium or one drachm of laudanum). (2) If the quantity of the opium or morphine ingested is unknown, eight, ten, or fifteen grains of the potassium salt, diluted in one pint of water, should be given, and repeated twice or three times at intervals of thirty minutes. In cases of poisoning by opium (laudanum), a teaspoonful of diluted sulphuric acid or white vinegar—preferably, diluted sulphuric acid—can be advantageously added to the antidotal solution. (3) Hypodermic injections of a well-diluted permanganate solution should be administered at short intervals. One grain in one ounce of water, injected with an anti-toxin syringe or an aspirator, will probably give better results than the use of concentrated solutions. (4) One grain of the antidote dissolved in a cupful of water should be given from time to time, in order to decompose the morphine, which the glandular lining of the stomach continues secreting for many hours.

But Sharp's[5] series of experiments showed that on mixing permanganate of potassium and morphine in solution the former decomposed or destroyed only about 94 per cent. of the morphine. True, he found that shortly after the administration of the permanganate in rabbits there was a marked rise in the number of respirations, showing it to influence in some manner physiologically, directly or indirectly, the respiratory mechanism, this being either by a stimulant or by an irritant action, and further that there is evidently some influence on the vaso-motor system due to some effect on the vaso-dilators or to vaso-constrictor paresis. Further, he concludes that the permanganate seems to act antagonistically to morphine when introduced hypodermically as it does when given by the stomach; but he does not think that there is sufficient evidence in cases reported to warrant the placing of any reliance on the permanganate solely as an antidote, and he thinks that its alleged properties are far from being established. On the other hand, from the experiment of Moor, the experiments of Wood, and one of his own self, he states that it would seem that the permanganate, when given with or immediately after the administration of the morphine, either by the stomach or as filtrate hypodermically, acts as a reliable antidote, and he feels warranted in believing that it is a valuable adjunct to the old treatment, but not reliable *per se*.

The Editor of the *Therapeutic Gazette*[6] points out that, as a matter of fact, a solution of permanganate of potassium injected into the subcutaneous tissues is at once oxidised and changed into a different substance, and can therefore no longer act as the permanganate; and even if this were not the case, the permanganate would have oxidised substances which it might have met with in the blood or other tissues of the body, and thus be unable to reach the morphine when widely distributed in various portions of the body. He, however, states that there is little doubt that this substance, when given by the mouth during the time that morphine still remains in the stomach, possesses distinct antidotal influence, since by its powerful oxidising properties it speedily destroys the alkaloid of opium.

[1] Lyon Méd., Sept. 8, 1895; Med. Chron., Nov., 1895. [2] N. Y. Med. Rec., Sept. 28, 1895. [3] Ther. Gaz., Oct. 15, 1895. [4] N. Y. Med. Rec., Oct. 26, 1895. [5] Loc. Cit. [6] Ther. Gaz., Oct. 15, 1895.

